# Ferroptosis: the emerging player in remodeling triple-negative breast cancer

**DOI:** 10.3389/fimmu.2023.1284057

**Published:** 2023-10-20

**Authors:** Jie Li, Dejiao He, Sicheng Li, Jun Xiao, Zhanyong Zhu

**Affiliations:** ^1^ Department of Thyroid and Breast Surgery, Shenzhen Qianhai Shekou Free Trade Zone Hospital, Shenzhen, China; ^2^ Department of Nephrology, Renmin Hospital of Wuhan University, Wuhan, China; ^3^ Department of Plastic Surgery, Renmin Hospital of Wuhan University, Wuhan, Hubei, China; ^4^ Department of Breast Surgery, Yueyang Central Hospital, Yueyang, Hunan, China

**Keywords:** breast cancer, ferroptosis, lipid, oxidative damage, tumor progression, immune therapy, therapy

## Abstract

Triple-negative breast cancer (TNBC) is a highly heterogeneous breast tumor type that is highly malignant, invasive, and highly recurrent. Ferroptosis is a unique mode of programmed cell death (PCD) at the morphological, physiological, and molecular levels, mainly characterized by cell death induced by iron-dependent accumulation of lipid peroxides, which plays a substantial role in a variety of diseases, including tumors and inflammatory diseases. TNBC cells have been reported to display a peculiar equilibrium metabolic profile of iron and glutathione, which may increase the sensitivity of TNBC to ferroptosis. TNBC possesses a higher sensitivity to ferroptosis than other breast cancer types. Ferroptosis also occurred between immune cells and tumor cells, suggesting that regulating ferroptosis may remodel TNBC by modulating the immune response. Many ferroptosis-related genes or molecules have characteristic expression patterns and are expected to be diagnostic targets for TNBC. Besides, therapeutic strategies based on ferroptosis, including the isolation and extraction of natural drugs and the use of ferroptosis inducers, are urgent for TNBC personalized treatment. Thus, this review will explore the contribution of ferroptosis in TNBC progression, diagnosis, and treatment, to provide novel perspectives and therapeutic strategies for TNBC management.

## 1 Introduction

Breast cancer is an increasingly prominent type of female tumor that contributes significantly to the disease burden worldwide. Triple-negative breast cancer (TNBC) is a highly heterogeneous breast tumor type that is highly malignant, invasive, and highly recurrent (1). TNBC is molecularly defined as a breast cancer subtype that is estrogen receptor (ER)-negative, progesterone receptor (PR)-negative, and does not overexpress human epidermal growth factor receptor 2 (HER2). Despite advances in basic research and treatment of TNBC, the combination of untimely diagnosis, lack of appropriate endocrine therapy and targeted therapy, and the aggressive nature, has led to poor clinical outcomes in TNBC (2). Currently, radiotherapy, immunotherapy, and targeted therapy, are profoundly affecting the clinical landscape of TNBC (3). Seeking more precise and effective therapeutic targets will provide a promising strategy for the personalized treatment of TNBC.

Programmed cell death (PCD) is an essential biological procedure in a variety of physiological circumstances, including cell fate, immune regulation, homeostasis of tissues and organs, and host immune defense against pathogens (4). The main types of PCD that have now been reported include apoptosis, autophagy, pyroptosis, necroptosis, ferroptosis, and the newly proposed cuproptosis. The novel concept of ferroptosis, first proposed by Dr. Brent R. Stockwell in 2012, is a new type of iron-dependent regulated cell death (5). Mechanistically, ferroptosis can cause cell death by loss of cell membrane integrity, lipid cross-linking that interferes with normal cell membrane function, and oxidative damage to macromolecules and cellular structures (6). Increased iron accumulation, free radical production, fatty acid supply, and lipid peroxidation are the key factors that induce ferroptosis (7). Ferroptosis is primarily realized through two major pathways: the exogenous pathway, initiated by inhibition of cell membrane transporter proteins such as cystine/glutamate reverse transporter protein (System xc-), or activation of iron transporter proteins; and the endogenous pathway, activated by blocking intracellular antioxidant enzymes (8). Ferroptosis is regulated by multiple cellular metabolic pathways, including redox homeostasis, iron metabolism, mitochondrial activity, and metabolism of amino acids, lipids, and sugars, as well as a diverse range of key disease-associated molecules.

Ferroptosis is critical in the progression of breast cancer, and induction of ferroptosis may improve the efficacy of tumor therapy. Multiple ferroptosis-associated regulators, such as glutathione (GSH), glutathione peroxidase 4 (GPX4), NFE2L2, superoxide dismutase (SOD), lipoxygenase, and coenzyme Q, are capable of remodeling breast cancer progression (9). Some key indicators of ferroptosis, such as acyl-CoA synthetase long-chain family 4 (ACSL4) and GPX4, their individual expression, and their combined status were independent prognostic factors for disease-free survival, demonstrating the diagnostic value of ferroptosis genes in breast cancer (10). Especially, TNBC cells have been reported to display a peculiar equilibrium metabolic profile of iron and GSH, which may increase the sensitivity of TNBC to ferroptosis (11). TNBC may exhibit higher sensitivity to ferroptosis than other breast cancer subtypes (12). Besides, ferroptosis inducers, such as GPX4 inhibitors, are available for treating TNBC (13). Therefore, ferroptosis is a crucial orchestrator in TNBC progression.

Based on the above observations, ferroptosis plays an important regulatory role in TNBC and is manifested by the fact that ferroptosis may be more likely to occur in tumors that are resistant to conventional therapy or have a high propensity to metastasize. The characteristic expression changes of ferroptosis-related genes may contribute to the construction of TNBC risk models. The immune effect of ferroptosis and therapeutic strategies in combination with other tumor treatments possess positive clinical value and significance. Thus, this review will explore the contribution of ferroptosis in TNBC progression, diagnosis, and treatment, to provide novel perspectives and therapeutic strategies for TNBC management.

## 2 The mechanism of ferroptosis and iron homeostasis

The critical molecular mechanism of ferroptosis involves regulating the balance between oxidative damage and antioxidant defense (14). In terms of biochemistry, ferroptosis is characterized by two main features, iron accumulation, and lipid peroxidation. Fe3+ bound to transferrin (TF) in serum is identified by transferrin receptor (TFRC) on the cell membrane, which in turn translocates it into the cell, while the solute carrier family 40 member 1 (Slc40a1) is in charge of transporting excess intracellular iron out into the extracellular space (15). Fe3+ entering the cell is reduced to Fe2+ by metal reductases, and Fe2+ plays an important role in metabolic and biochemical processes in mitochondria. Excess iron can also be stored by binding to ferritin, which can be lysosomally degraded to increase intracellular free iron levels, thus inhibiting the ferritin (FTH) degradation process would limit ferroptosis (16).

Intracellular iron accumulation is a key factor in ferroptosis, attributed to several fundamental processes of cellular metabolism. Iron is required to catalyze some important enzymes in phospholipid peroxidation, such as lipoxygenases (LOX) and cytochrome P450 reductase (POR), as well as many metabolic enzymes involved in intracellular reactive oxygen species (ROS) generation (17). Second, when GPX4 is inhibited, phospholipid hydroperoxide (PLOOH) amplified by the iron-dependent Fenton reaction can react with iron ions to produce free radicals and further amplify this process, which is essential in iron apoptosis (8).

Lipid peroxidation is a free radical-driven reaction, which primarily influences unsaturated fatty acids in cell membranes. Intracellular polyunsaturated fatty acids (PUFA) are catalyzed to produce phospholipid-polyunsaturated fatty acids (PL-PUFA), which can be peroxidized either enzymatically or nonenzymatically to form PLOOH (18). If PLOOH is not neutralized in a timely manner after its formation, it can propagate its peroxidation to neighboring PUFA-PL in the presence of unstable iron, facilitating the ferroptosis progress (19).

**Figure 1:**
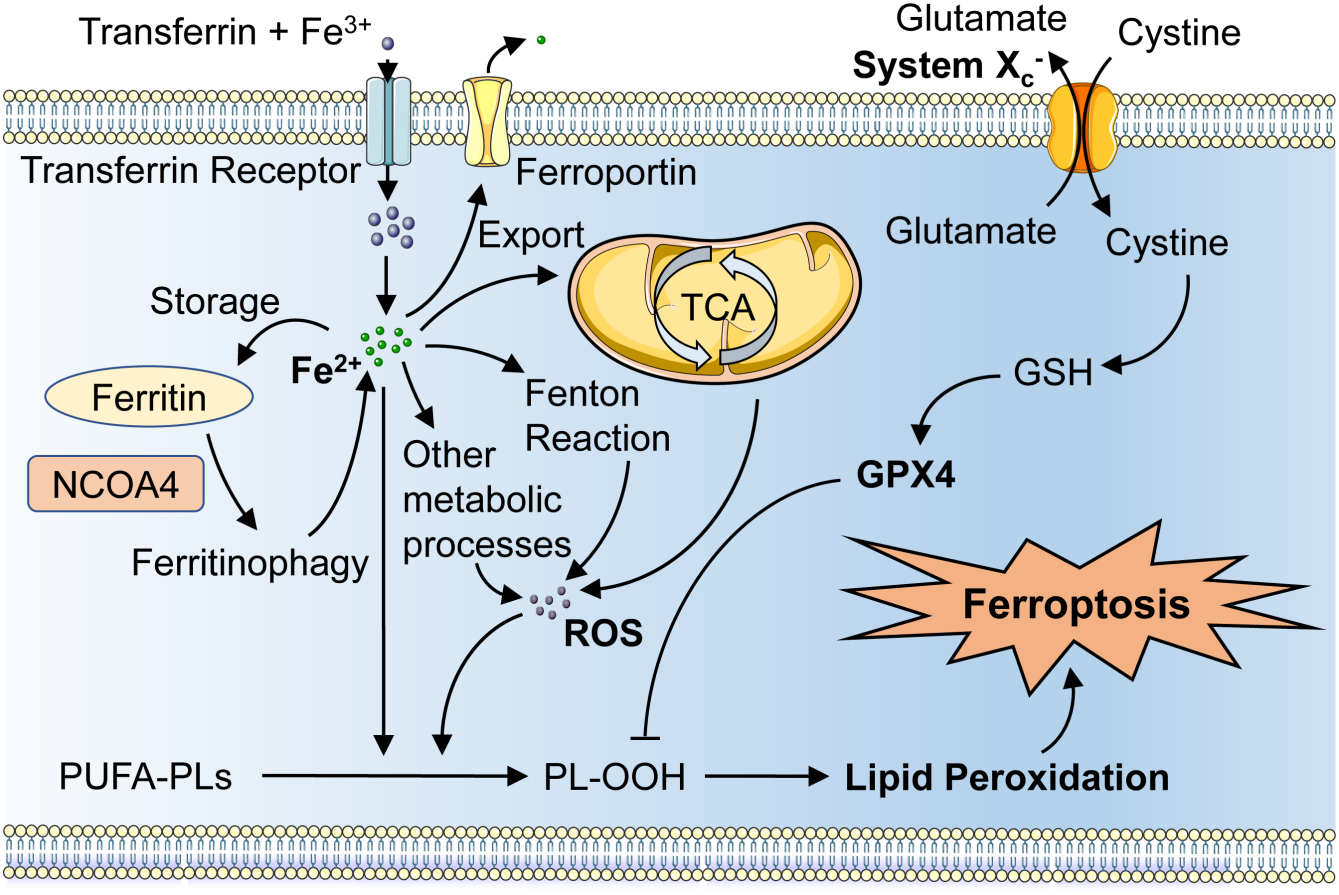
Schematic representation of the mechanism of ferroptosis. Fe3+ imported through the transferrin and TFRC could convert to Fe2+ and increase iron accumulation, and excess iron can be stored by ferritin or exported by ferroportin. NCOA4 binds to ferritin resulting in ferritinophagy, which then increases the intracellular free iron. The tricarboxylic acid cycle and Fenton’s reaction as well as other metabolic processes can System Xc- can increase cystine and glutamate transport, which leads to the suppression of lipid peroxidation by GPX4 and thus inhibit iron oxidation. TFRC, transferrin receptor; GPX4, glutathione peroxidase 4; NCOA4, nuclear receptor coactivator 4.

Since ferroptosis is a result of an imbalance between oxidative and antioxidant oxidation in the cell, intracellular antioxidant factors, such as the GPX4 antioxidant system, are available to protect the cell from ferroptosis. System Xc- is an important part of the antioxidant system in the cell, which is responsible for the transport of cysteine and glutamate in and out of the cell (20). Uptaken cysteine can be reduced to cysteine, which then participates in the synthesis of GSH. GPX4 could terminate phospholipid peroxidation by converting GSH to oxidized glutathione (GSSG) and catalytically reducing toxic PLOOH to nontoxic PL alcohols (PLOH) (21). This is a classical pathway that protects cells from ferroptosis, and the inhibition of system Xc- and GPX4 activities could lead to impaired cellular antioxidant capacity and the accumulation of lipid peroxides, which consequently promote ferroptosis (**Figure 1**).

## 3 Ferroptosis-related proteins in TNBC progression

Cannabinoid receptor 1 (CB1) is an important category of G protein-coupled receptors involved in mood regulation, cognitive function, and cell fate regulation (22). It is also a potential target for the treatment of a variety of neurological disorders and obesity. Li et al. indicated that the CB1 antagonist rimonabant potentiated erastin/RSL3-induced iron metabolism as evidenced by the enhancement of lipid peroxidation, malondialdehyde (MDA), 4-hydroxynonenal (4-HNE), and ROS, and ultimately potentiated its inhibitory effect on tumor proliferation (23). CB1 could regulate SCD1- and FADS2-dependent fatty acid metabolism to regulate ferroptosis sensitivity in TNBC. These suggest that dual targeting of CB1 and ferroptosis sensitivity may be an efficient synergistic therapeutic strategy for treating TNBC. Compared to other breast cancer subtypes, progesterone receptor membrane component-1 (PGRMC1) was the highest expressed in TNBC. PGRMC1 could act as an iron-binding protein to reduce intracellular iron ion concentration, inhibit ferroptosis, and foster TNBC malignancy (24).

**Figure 2:**
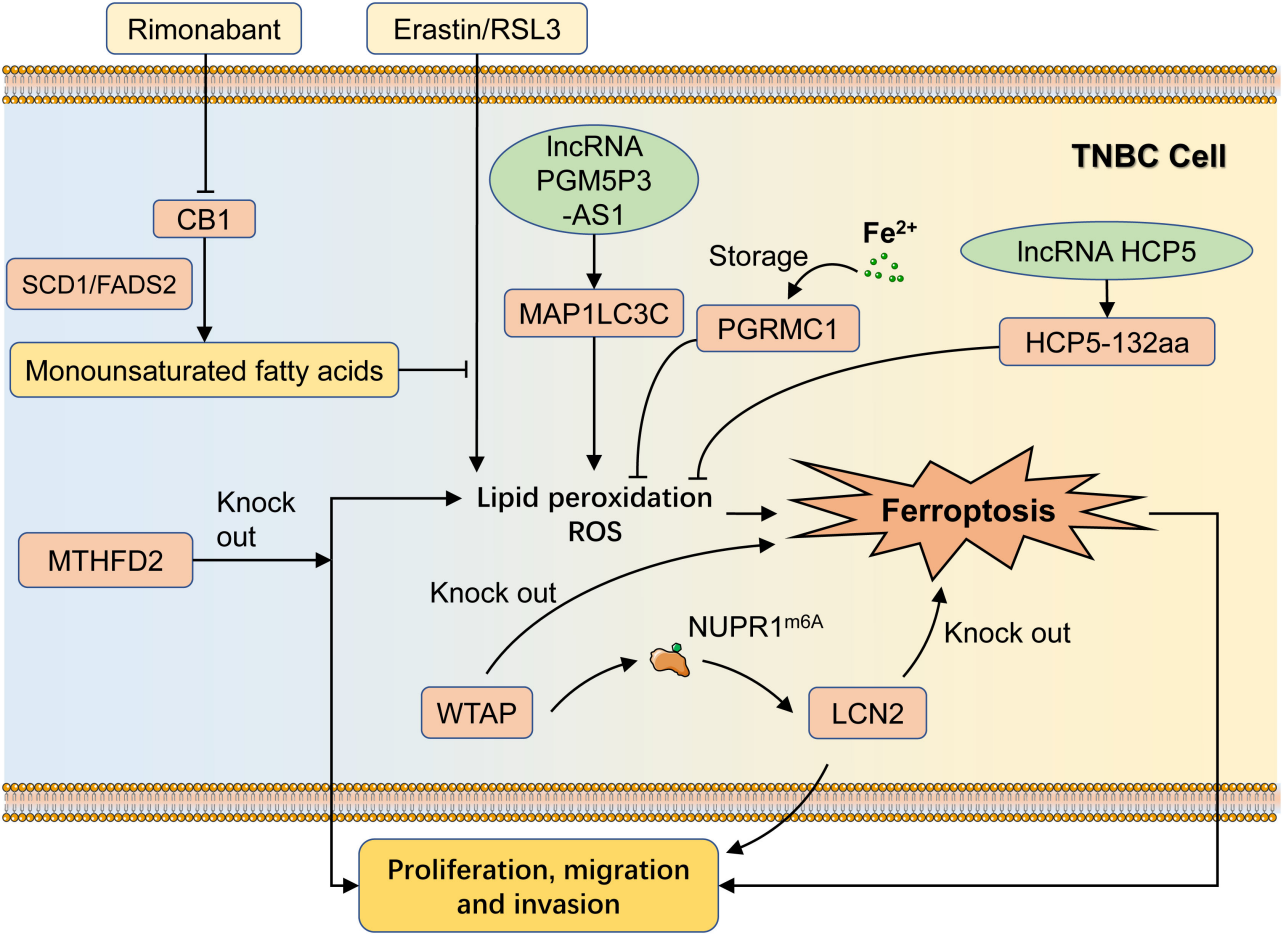
Regulatory mechanisms of ferroptosis in TNBC progression. CB1 can promote the production of monounsaturated fatty acids in an SCD1/FADS2-dependent manner and then suppress the erastin/RSL3-induced ferroptosis, while the CB1 inhibitor Rimonabant could inhibit this process. PGRMC1 can bind with intracellular free iron and reduce the concentration of free iron, leading to the inhibition of ferroptosis. MTHFD2 knockdown induces ferroptosis in TNBC cells as well as inhibits TNBC progression. WTAP can promote LCN2 upregulation via NUPR1m6A, leading to TNBC progress. LncRNA HCP5 can suppress ferroptosis in TNBC cells by encoding the protein HCP5-132aa, while PGM5P3-AS1 can promote ferroptosis as a result of regulating MAP1LC3C. TNBC, Triple-negative breast cancer; RSL3, ras-selective lethal 3; PGRMC1, progesterone receptor membrane component-1; MTHFD2, methylenetetrahydrofolate dehydrogenase 2; Lcn2, lipocalin 2.

Methylenetetrahydrofolate dehydrogenase 2 (MTHFD2) was highly expressed in TNBC and negatively correlated with prognosis (25). MTHFD2 knockdown suppressed biological effects of TNBC cells, such as proliferation and invasion. In addition, MTHFD2 knockdown cells suffered from ferroptosis and exhibited remarkable up-regulation of intracellular ROS, lipid peroxidation, and MDA levels, which led to tumor cell impairment. Therefore, it hypothesized that MTHFD2 was the potential gene for regulating ferroptosis in TNBC. Wilms’ tumor 1-associating protein (WTAP) is necessary for m6A modifications and modulates multiple cellular processes, including RNA splicing, gene expression, and cell behaviors (26). WTAP affected ferroptosis by modulating the modification of m6A in NUPR1 and thus positively upregulating lipocalin 2 (Lcn2), thereby promoting breast cancer proliferation, migration, and invasion (27). Knockdown of either WTAP or LCN2 could induce ferroptosis, thereby inhibiting the malignant behavior of TNBC cells, demonstrating the role of WTAP-mediated m6A and LCN2 expression in TNBC ferroptosis (**Figure 2**).

## 4 LncRNAs in ferroptosis of TNBC

LncRNA is a class of non-coding RNAs greater than 200 nt in length that do not translate proteins and is extensively found in a variety of animal and plant cells. LncRNAs are involved in a variety of biological regulatory processes, such as chromatin remodeling, variable shearing, and translational regulation, and specific lncRNAs are also capable of encoding protein. Tong et al. demonstrated that the ORF of lncRNA HCP5 could encode a 132 amino acid protein, denoted as HCP5-132aa, and that its high expression was strictly associated with poorer prognosis (28). Biologically, overexpression of HCP5-132aa was able to aggravate the TNBC process and intervene in the ferroptosis process by regulating GPX4 expression and lipid ROS levels. In addition, PGM5P3-AS1, a low-expressed lncRNA in TNBC cells, promoted cellular ferroptosis and inhibited TNBC progression by regulating MAP1LC3C (29) (**Figure 2**).

## 5 Ferroptosis in immune regulation of TNBC

TNBC exhibits a heterogeneous phenotype in terms of metabolic pathways associated with ferroptosis. The therapeutic application of programmed cell death protein 1 (PD-1)/PD-L1 inhibitors for the immunomodulation and treatment of TNBC is currently a promising immunotherapeutic strategy (30). Yang et al. showed that the luminal androgen receptor (LAR) subtype of TNBC was featured by upregulated oxidized phosphatidylethanolamine and GSH metabolism, specifically GPX4 (31). Thus, GPX4 inhibitors could induce ferroptosis, and targeting GPX4 might be a feasible therapeutic strategy. Clinically, higher GPX4 expression was associated with lower cytolysis scores and poorer prognosis for immunotherapy (31). Combination therapy with GPX4 inhibitors and anti-PD1 resulted in better tumor suppression and remodeling of tumor microenvironment (TME) compared with monotherapy, suggesting that the combination strategy could be effective in treating tumors with similar biological characteristics to LAR. Wang et al. showed that protein arginine methyltransferase 5 (PRMT5) was repressed ferroptosis in TNBC cells and similarly enhanced ferroptosis tolerance by inhibiting the NRF2/HMOX1 pathway by methylating and stabilizing KEAP1 (32). High levels of PRMT5 protein suggested that TNBC were highly resistant to immunotherapy, whereas PRMT5 inhibitors were able to enhance the efficacy of immunotherapy, such as using anti-PD-1 antibodies. Thus, PRMT5 might regulate iron metabolism, and act as an intervention target for TNBC immunotherapy tolerance.

Macrophages are a heterogeneous population of functionally complex and phenotypically diverse cells. Tumor-associated macrophages (TAMs) are of the M2 type to induce immune inhibition, achieved by secretion of regulatory cytokines and inhibition of tumor-infiltrating Lymphocytes (TILs) (33). Li et al. demonstrated that hepatic leukemia factor (HLF) could transactivate the expression of gamma-glutamyltransferase 1 (GGT1), and facilitate ferroptosis tolerance, which was responsible for potentiating malicious behaviors and cisplatin resistance of TNBC cells (34). As an oncogenic protein, HLF was modulated by TAM-derived TGF-β1, while TNBC-derived IL-6 activated the JAK2/STAT3 axis to induce TAMs-derived TGF-β1, thus forming a feedback loop. This effect resulted in sustained activation of HLF and enhanced ferroptosis tolerance in TNBC cells. Therefore, HLF could be regarded as a therapeutic target for ferroptosis-associated tumor therapy.

**Figure 3:**
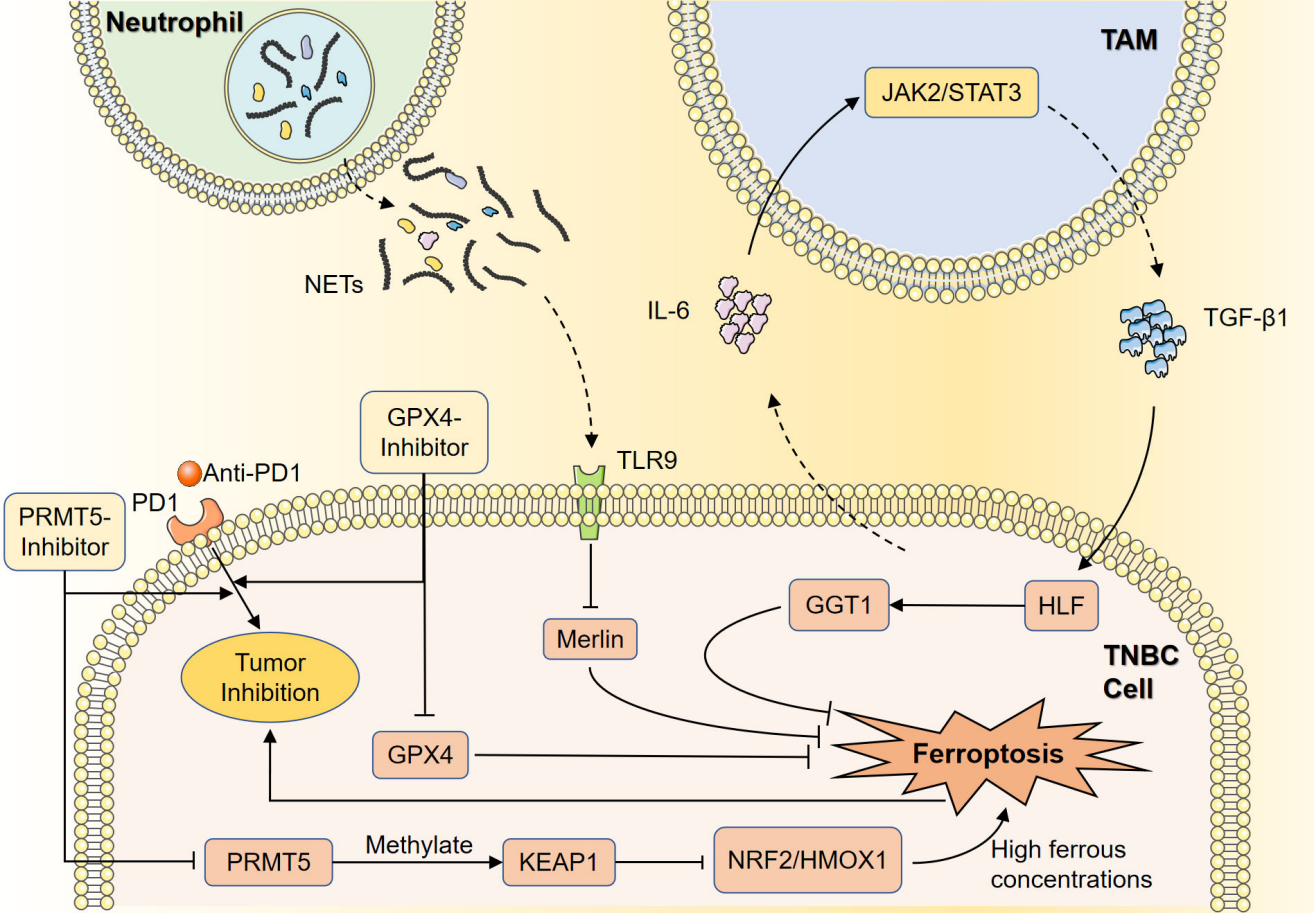
Ferroptosis in immune regulation of TNBC. Anti-PD1 therapy is an important part of immunotherapy, and GPX4 inhibitors may enhance the tumor-suppressive effects of anti-PD1 therapy by inducing ferroptosis. PRMT5 can methylate KEAP1 to inhibit the NRF2/HMOX1 pathway, resulting in ferroptosis suppression in the presence of high intracellular ferrous concentrations. PMRT5 inhibitors could prevent this process and improve the efficacy of immunotherapy. TAMs can regulate HLF by secreting TGF-β1, while HLF can suppress ferroptosis in TNBC cells through activation of GGT1. TNBC cells can activate the JAK2/STAT3 axis by secreting IL-6, resulting in the secretion of TGF-β1 by TAMs and the sustained activation of HLF. NETs can downregulate the phosphorylation of Merlin by interacting with TLR9, thus inhibiting ferroptosis in TNBC cells. TNBC, Triple-negative breast cancer; GPX4, glutathione peroxidase 4; PD-1, programmed cell death protein 1; GGT1, gamma-glutamyltransferase 1; HLF, hepatic leukemia factor; NETs, neutrophil extracellular traps; TLR9, toll-like receptor 9; TAMs, tumor-associated macrophages.

Neutrophil extracellular traps (NETs) are key immune cell components that promote TNBC tumor progression and lymph node metastasis. In experimental validation, NET was found to promote TNBC cell proliferation and lung metastasis. This was because NET could interact with toll-like receptor 9 (TLR9), leading to down-regulation of Merlin phosphorylation, which promoted the phenomenon of ferroptosis tolerance (35). It was hypothesized that blocking NETs and related pathways might contribute to TNBC therapy. Therefore, targeting the regulation of ferroptosis in these immune cells is a potential therapeutic strategy to reverse immunosuppressive TME of TNBC (**Figure 3**).

## 6 Ferroptosis in TNBC diagnosis

Due to the high heterogeneity of TNBC and its association with specific gene mutations, single pathologic indicators or molecular markers are not effective enough for the diagnosis and recurrence evaluation of TNBC. Many ferroptosis-related genes or molecules have characteristic up- or down-regulated expression patterns and are expected to be diagnostic targets for TNBC. In particular, more biologically relevant information on these markers, including biological processes, immunomodulatory correlations, and drug resistance analysis, can be discovered by mining open-source databases such as The Cancer Genome Atlas (TCGA) and Gene Expression Omnibus (GEO). Therefore, prediction models constructed based on a single marker or multiple markers may play a very important role in the prognostic judgment and accurate grading of TNBC.

For example, Li et al. established a risk model to assess the prognosis of TNBC patients, based on iron metabolism and immunity-related genes, including EGR3, SOCS3, JunD, SLC27A6, and CCND2 (36). Categorized into high and low risk groups based on model scores, patients in the low risk group had a better prognosis, accompanied by accurate AUC values and excellent predictive ability. The low risk group was associated with higher tumor immune infiltration and abundance. Therefore, the model can reflect TNBC prognosis, and molecular characteristics with some degree of accuracy and be used to guide treatment. However, the study was mainly based on the existing TCGA and GEO databases, and there is a lack of external data cohorts and experimental validation, which is necessary to improve the accuracy of the model. Yuan et al. constructed the expression patterns of ferroptosis-related genes and found that 7 genes, such as CA9, CISD1, STEAP3, HMOX1, DUSP1, TAZ, and HBA1, were closely related to the overall survival (OS) of TNBC (37). In particular, STEAP3 showed high expression in TNBC tissues and cells. High levels of STEAP3 expression have a detrimental effect on OS in TNBC patients and were predictive of the response of TNBC patients to chemotherapy. Fang et al. identified and constructed a prediction model consisting of 12 ferroptosis-related genes through public database mining (38). The model had a high AUC and functioned as an independent predictor. The screened-out 7 genes, including ASNS, LAMP2, CAV1, DPP4, HELLS, TF, and ZFP69B, were potential targets for TNBC treatment. By using the CTD database, rosiglitazone and 1-methyl-3-isobutyl-cryptoxanthine were found to be potential inducers of ferroptosis-related drugs.

Additionally, Wu et al. constructed and identified 15 ferroptosis-related gene models for TNBC prediction (39). The models were able to accurately differentiate the prognosis of high- and low-risk patients and indicated the cellular and molecular features of the tumor immune microenvironment. Fang et al. also constructed a prognostic prediction model for TNBC. After being categorized according to the model score, TNBC patients with low-risk scores experienced higher OS and better prognosis (40). Meanwhile, in immunotherapy, patients with high-risk scores had higher responsiveness to anti-PD-1 blockers or sunitinib and better treatment outcomes. Therefore, this model was able to distinguish the risk of prognosis and the benefit of immunotherapy to some extent, further confirming the predictive possibility and high efficiency of ferroptosis-related genes. Li et al. intended to develop a signature based on 9 FRGs that could be used to predict disease-specific survival (DSS) in BC patients (41). The risk ratings delineated by this signature were tightly associated with tumor staging grades, TNBC subtypes, and the benefit of CTLA4 and PD-1-targeted therapies.

## 7 Ferroptosis in TNBC treatment

### 7.1 Ferroptosis in TNBC drug resistance

Many existing chemotherapeutic agents can induce ferroptosis and dysregulation of oxidative stress in ferroptosis can lead to chemotherapeutic drug resistance and poor treatment. Currently, targeting ferroptosis can reverse chemotherapeutic resistance, which mainly consists of three pathways related to the ferroptosis mechanism, typically the GPX4-regulated pathway, the iron metabolism pathway, and the lipid metabolism pathway (42). Song et al. demonstrated that GPX4 was overexpressed in gefitinib-resistant MDA-MB-231/Gef and HS578T/Gef cells (43). *In vitro*, silencing GPX4 promoted ferroptosis to increase gefitinib sensitivity. Liang et al. indicated that targeted inhibition of heat shock protein beta-1 (HSPB1) was a key therapeutic strategy for BC inhibition and resistance to chemotherapy (44). Clinical and pathological data showed that HSPB1 was a highly expressed and pro-carcinogenic gene capable of promoting doxorubicin (DOX) resistance by protecting against DOX-induced ferroptosis in tumor cells. Mechanistically, HSPB1 could bind to Ikβ-α and promote its ubiquitination-mediated degradation, thereby causing increased nuclear translocation and activation of NF-κB signaling.

Radiation could induce the death of MDA-MB-231 cell line, and enhance the iron level and ROS production (45). Iron deposition and iron-regulated proteins, including TF, TFRC, and FTH, were observed to increase following radiation exposure, indicating the occurrence of ferroptosis. Radiation-induced iron accumulation increased ROS levels via the Fenton reaction and increased autophagy. Therefore, radiation-induced iron-dependent autophagic cell death in TNBC cells was a central event, underlying the development of sensitization strategies for cell death tolerance. Sun et al. reported the effects of 3 propofol formulations on anti-TNBC tumor effects, including propofol, propofol injectable emulsion (PIE), or fospropofol disodium (46). The results showed that these propofol formulations potentiated the antitumor effects of DOX and paclitaxel by enhancing apoptosis, elevated intracellular ROS levels, and ferroptosis, a process that was implicated in alterations in the p53-SLC7A11-GPX4 pathway. This phenomenon gave evidence that propofol improved the chemosensitivity of TNBC cells by promoting ferroptosis.

### 7.2 Ferroptosis inducers in TNBC treatment

Ferroptosis is a common mechanism of cell death induced by frequently used antitumor drugs. The strategies based on death mechanisms, drug repositioning, and subgroup discovery, are urgent for TNBC personalized treatment (47). Notably, natural products are an important source of drug development and have shown strong and multifaceted positive effects in inflammation, infection, tumor, and neurological disorders. Several natural products have been identified with the properties of inducing ferroptosis, sensitizing tumor therapeutic effects, and inhibiting tumor growth, such as artemisinin (48). Tapping new chemically synthesized drugs or naturally isolated drugs by interfering with the ferroptosis mechanism contributes to tumor drug development and synergistic therapeutic strategies (49). Currently, several relevant drugs based on ferroptosis have been used in cellular and animal therapeutic studies of TNBC.

Iron-saturated Lf (Holo-Lactoferrin, Holo-Lf) is a natural protein that represents a potential ligand for the TFRC (50). Zhang et al. emphasized that Holo-Lf promoted iron content in MDA-MB-231 cells, promoted ROS and MDA production, and thus enhanced ferroptosis (51). Meanwhile, Holo-Lf downregulated the expression of HIF-1α, and increased the sensitivity of radiotherapy. Zhao et al. chemically synthesized a derivative of curcuminol, HCL-23, and validated its TNBC therapeutic mechanism by sequencing and experiments (52). HCL-23 was able to provoke ferroptosis by increasing levels of ROS, labile iron pool (LIP), and lipid peroxidation, and accordingly inhibited TNBC growth *in vivo*. This cell death effect of HCL-23 in TNBC was mediated by caspases-associated apoptosis and heme oxygenase-1 (HO-1)-dependent ferroptosis. A natural compound, hinokitiol (hino), could trigger apoptosis in TNBC cells (53). The hino, acting as an iron chelator, could complex with iron to form Fe(hino)3, the latter of which was recognized to exhibit redox activity that facilitated the production of free radicals via the Fenton reaction. Thus, Fe(hino)3 was identified as a ferroptosis inducer, demonstrating promising therapeutic effects in TNBC at both cellular and animal.

Several compounds isolated from natural medicines demonstrated different mechanisms and pathways of ferroptosis induction to exert therapeutic effects on TNBC. Wei et al. isolated a natural compound eupaformosanin (Eup) from *Eupatorium cannabinum Linn.*, and found that it significantly inhibited the proliferation of TNBC cells (54). This function occurred through ubiquitination of mutant p53, and the mutant p53 signaling pathway was involved in Eup-induced apoptosis and ferroptosis. Thus, Eup conferred the inhibitory effects by inducing apoptosis and ferroptosis in TNBC. A derivative of the natural product parthenolide, DMOCPTL, possessed robust anti-TNBC tumor activity (55). In addition, GPX4 was an overexpressed protein in TNBC tissues, and GPX4 induced apoptosis through upregulating EGR1 in TNBC cells. DMOCPT primarily executed its anti-tumor function by directly binding to GPX4 protein and promoting GPX4 ubiquitination, which in turn induced ferroptosis and apoptosis. *In vivo* studies also demonstrated that DMOCPTL suppressed TNBC and prolonged survival in mice with a high biosafety. Anomanolide C (AC), a natural withanolide isolated from *Tubocapsicum anomalum*, AC was capable of inducing autophagy and ferroptosis and could induce autophagy-dependent FTH deposition by ubiquitinating GPX4 (56). This effect resulted in a potent inhibitory effect of AC on TNBC progression and metastasis. Tiliroside (Til), an effective natural flavonoid glycoside of the oriental paperbush flower, has been shown to possess anticancer effects in many types of tumors. Hu et al. reported that Til can exert antitumor activity via the PUFA-PLS pathway by promoting ferroptosis in TNBC cells, achieved by the HO-1/SLC7A11 pathway (57). Shuganning injection (SGNI), a traditional Chinese patent medicine, could trigger the ferroptosis effect to inhibit TNBC cell proliferation and tumor growth. This result was inhibited by ferroptosis inhibitors such as ferrostatin-1 and was closely related to HO-1 function (58). Liu et al. developed a germacranolide So-2 from the aerial parts of *S. orientalis*, which exhibited potent cycle blocking and inhibition of TNBC cells (59). In addition, So-2 induced TNBC ferroptosis by downregulating E2F7 expression, which led to suppressive properties of TNBC *in vitro* and *in vivo*. This result suggested that So-2, a natural compound isolated from traditional Chinese medicine, might be a potential ferroptosis-related therapeutic agent for combating TNBC (**Table 1**).

**Table 1:** The mechanisms of emerging ferroptosis inducers in TNBC treatment.

Molecule	Resource	Mechanism	Effect	Reference
Holo-Lf	Human	Downregulated GPX, GSH, HIF-1α expression, and ameliorated hypoxia microenvironment	Enhanced ferroptosis and radiosensitivity of MDA-MB-231 cells and tumors	(51)
HCL-23	*Curcumol*	Upregulated caspase family expression and HO-1 expression	Inhibited TNBC cell proliferation and metastasis, induced cell cycle arrest, apoptosis, and ferroptosis both in vitro and in vivo	(52)
Fe(hino)3	*Thuja Plicata*	Induced GSSG/GSH depletion and boosted iron-dependent lipid peroxidation	Induced TNBC cell ferroptosis, suppressed breast cancer-derived lung metastasis, inhibited tumor growth	(53)
Eup	*Eupatorium*	Downregulated caspase 3 expression, disrupted mitochondrial membrane potential, and promoted mutant p53 ubiquitination	Suppressed the growth of MDA-MB-231-driven tumors, induced apoptosis and ferroptosis, and reduced mutant p53 level	(54)
DMOCPTL	*Tanacetum parthenium*	Induced the release of cytochrome C and triggers the cleavage of caspase 9, caspase 3, and PARP, promoted GPX4 ubiquitination and upregulated EGR1 expression	Induced mitochondria-mediated apoptosis and ferroptosis of TNBC cells,inhibited growth of TNBC and prolong survival life of mice in vivo	(55)
Anomanolide C	*Tubocapsicum anomalum*	Decreased FTH1, GPX4, SLC7A11 expression	Induced autophagy-dependent ferroptosis and inhibited proliferation and migration in TNBC cells	(56)
Tiliroside	*Edgeworthia chrysantha*	Promoted Nrf2 and HO-1 expression	Induced HO-1 dependent ferroptosis and exerted antitumor activity on TNBC	(57)
SGNI	*Ganoderma lucidum, Isatidis Radix, Gardeniae Fructus, Artemisiae Scopariae Herba*	Induced cellular oxidative stress and HO-1 mediated Fe^2+^ accumulation	Induced ferroptosis in TNBC cells and inhibited tumor growth both in vitro and in vivo	(58)
So-2	*Siegesbeckiaorientalis L.*	Downregulated E2F7 expression	Inhibited proliferation and migration, promoted cell cycle arrest in G2/M phase and ferroptosis in TNBC cells	(59)

## 8 Discussion

Breast cancer cells are phenotypically plastic and highly heterogeneous, making them highly adaptive and treatment-resistant, successfully circumventing cell death regulation (60). Ferroptosis is a specific form of cell death with unique mechanisms and characteristics compared to other forms of PCD. TNBC have a higher sensitivity to ferroptosis than other breast cancer types. Therefore, induction of ferroptosis may be a potential strategy to optimize TNBC treatment. There are still some issues to consider in this field.

First, the elucidation of the mechanism of ferroptosis for TNBC presented in the current study is still limited. In the complex biological system of TNBC, ferroptosis effect is regulated by multiple biological mechanisms, including epigenetics, PTM modification, and immune behavior regulation. Ferroptosis often have some cross-correlation with other regulatory mechanisms. Different PCD types possess different molecular mechanisms and death modalities, and these PCD modalities interact and influence each other. For example, autophagy may mediate FTH catabolism and increase iron and ROS, while excess ROS activates autophagy and remodels cell fate and chemoresistance. Autophagy promotes ferroptosis by degradation of FTH in fibroblasts and cancer cells, demonstrating the co-existence and mutual regulation of autophagy and ferroptosis in stromal and tumor cells (61). NLRP3 inflammasome, a classical indicator of pyroptosis, whose activation not only contributes to pyroptosis but also has inextricable effects on apoptosis, necroptosis, and ferroptosis (62). Due to the heterogeneity of TNBC, the molecular mechanism is still a mystery, and whether there are other ferroptosis-regulating genes remains to be thoroughly investigated. Corresponding genes and molecules may facilitate the excavation of novel ferroptosis inhibitors or inducers.

Ferroptosis-related genes and pathway proteins may have spatiotemporal specific changes in the course of breast cancer. These markers with altered expression may respond at the level of the tumor *in situ* or as markers for hematological tests. Existing studies have reported that a variety of risk models or signatures are constructed based on ferroptosis-related genes and have shown predictive efficacy in characterizing the immune milieu, prognosis, and therapeutic benefit. Therefore, assessing the susceptibility or efficacy of ferroptosis therapies or predicting adverse events by detecting the levels of specific biomarkers is a potentially effective strategy for breast cancer diagnosis. However, most of these single-gene or multigene modeling studies are based on already reported databases, including TCGA and GEO. That is, these predictors and models tend to be retrospective studies that lack more experimental validation and real-world data. The detection efficacy of these methods needs to be fully confirmed by prospective cohort studies. Besides, These models, constructed for ferroptosis-related genes, are a long way from clinical application. First, these novel models can only serve as a useful supplement to existing diagnostic modalities, but not as a replacement for the gold standard of blood, imaging and pathology tests. Second, these models have not been formally applied in clinical diagnosis, but have only been proposed as basic models for research. Finally, certain characteristic ferroptosis-related genes have not been analyzed by sufficient basic and clinical studies. The feasibility of these included genes for diagnostic use needs to be fully validated.

Targeting the pathway of ferroptosis in tumor cells to induce ferroptosis is an emerging antitumor strategy for the treatment of TNBC. Ferroptosis inducers have been studied to act directly on breast cancer cells to exert anticancer effects and also as sensitizers for radiotherapy to reverse tumor radiotherapy resistance. Ferroptosis inducers that have been investigated currently include Liuzosulfapyridine, Sorafenib, ras-selective lethal 3 (RSL3), and artemisinin derivatives. Ferroptosis inducers can be classified into 4 categories according to their targets, including System Xc-, targeting GSH, targeting GPX4, and targeting ferric ions and ROS. Ferroptosis occurs not only in TNBC, but additionally in stromal tissues, such as tumor-associated adipocytes, stromal cells, and immune cells. Different metabolic types between cancer cells and normal cells lead to different propensities, degrees, and outcomes of ferroptosis. Moreover, the signaling pathways regulating ferroptosis exist in as yet unclear crosstalk with many classical cell signaling pathways, such as the energy metabolism pathway with the PI3K-AKT-mTOR pathway (63). These factors, mentioned above, can lead to ferroptosis-based tumor therapies that may result in unexpected off-target and adverse effects. Therefore, continued research in this field needs to ground further search for ways to improve targeting against breast cancer cells to reduce the generation of side effects.

The development of a multifunctional nano-based therapeutic platform can effectively enrich the concentration of ferroptosis inducers, leading to superior therapeutic efficacy and safety. For example, simvastatin is commonly used to decrease blood lipid levels and is a well-established hydroxymethylglutaryl coenzyme A (HMG-CoA) reductase inhibitor (64). Simvastatin possesses certain antitumor activity because of its ability to promote the proliferation and cytotoxicity of tumor antigen-specific CD8+ T cells (65). Simvastatin inhibited the expression of HMGCR, and thus down-regulated the mevalonate pathway and GPX4 expression, thereby inducing ferroptosis in MDA-MB-231 cells (66). Compared with simvastatin alone, encapsulation of simvastatin into zwitterionic polymer-coated magnetic nanoparticles (Fe3O4@PCBMA) could promote MDA-MB-231 cell death by targeting accumulation and inducing ferroptosis. Yu et al. constructed an erastin-loaded exosome vehicle using folic acid (FA) (erastin@FA-exo) for targeting TNBC cells (67). *In vivo*, erastin@FA-exo facilitated ferroptosis, resulting in intracellular GSH depletion and ROS generation in excess. However, the system of nanomaterials is complex, and the pharmacokinetic characteristics and biosafety of the drugs need to be further investigated. Exploring combinations of ferroptosis-targeted therapy combined with other therapeutic approaches, such as immunotherapy and gene therapy, is also a feasible direction of development. Currently, most of these therapies are still at the cellular and animal levels, and their safety and efficacy on patients have not been conclusively demonstrated. Therefore, large-scale, multicenter clinical trials are still necessary to evaluate the safety and efficacy of targeted ferroptosis strategies in TNBC patients.

## 9 Conclusion

In summary, ferroptosis, as an iron-dependent PCD modality characterized by disturbed GSH metabolism and lipid peroxidation, plays an important role in the different stages of TNBC and in malignant biological behaviors, such as proliferation, invasion, and migration. Constructing a risk model related to ferroptosis is an effective adjunct to TNBC diagnosis, tumor infiltration scoring, and prognostic assessment. Finally, TNBC exhibits a distinctive expression pattern of ferroptosis-related genes, making it particularly susceptible to ferroptosis inducers. Developing therapeutic modalities that target ferroptosis and combining it with other therapeutic approaches is a promising strategy for the therapy of TNBC.

## Author contributions

JL: Writing – original draft, Writing – review & editing. DH: Writing – original draft, Writing – review & editing. SL: Writing – original draft, Writing – review & editing. JX: Conceptualization, Writing – original draft, Writing – review & editing. ZZ: Conceptualization, Funding acquisition, Writing – original draft, Writing – review & editing.

## Glossary

**Table:**

4-HNE	4-hydroxynonenal
ACSL4	acyl-CoA synthetase long-chain family 4
AC	anomanolide C
CB1	cannabinoid receptor 1
POR	cytochrome P450 reductase
DSS	disease-specific survival
DOX	doxorubicin
ER	estrogen receptor
Eup	eupaformosanin
FTH	ferritin
FA	folic acid
GGT1	gamma-glutamyltransferase 1
GEO	Gene Expression Omnibus
GSH	glutathione
GPX4	glutathione peroxidase 4
HSPB1	heat shock protein beta-1
HO-1	heme oxygenase-1-dependent ferroptosis
HLF	hepatic leukemia factor
hino	hinokitiol
HER2	human epidermal growth factor receptor 2
HMG-CoA	hydroxymethylglutaryl coenzyme A
Holo-Lactoferrin	Holo-Lf, iron-saturated Lf
LIP	labile iron pool
Lcn2	lipocalin 2
LOX	ipoxygenases
LAR	luminal androgen receptor
MDA	malondialdehyde
MTHFD2	methylenetetrahydrofolate dehydrogenase 2
NETs	neutrophil extracellular traps
NCOA4	nuclear receptor coactivator 4
OS	overall survival
PLOOH	phospholipid hydroperoxide
PL-PUFA	phospholipid-polyunsaturated fatty acids
PUFA	polyunsaturated fatty acids
PR	progesterone receptor
PGRMC1	progesterone receptor membrane component-1
PCD	programmed cell death
PD-1	programmed cell death protein 1
PIE	propofol injectable emulsion
PRMT5	protein arginine methyltransferase 5
RSL3	ras-selective lethal 3
ROS	reactive oxygen species
SGNI	shuganning injection
Slc40a1	solute carrier family 40 member 1
SOD	superoxide dismutase
TCGA	The Cancer Genome Atlas
Til	tiliroside
TAMs	tumor-associated macrophages
TLR9	toll-like receptor 9
TF	transferrin
TFRC	transferrin receptor
TNBC	triple-negative breast cancer
TME	tumor microenvironment
TILs	tumor-infiltrating Lymphocytes
WTAP	Wilms’ tumor 1-associating protein
